# Double blocking of carbon metabolism causes a large increase of Calvin–Benson cycle compounds in cyanobacteria

**DOI:** 10.1093/plphys/kiae083

**Published:** 2024-02-20

**Authors:** María Teresa Domínguez-Lobo, Miguel Roldán, Alba María Gutiérrez-Diánez, Francisco Javier Florencio, María Isabel Muro-Pastor

**Affiliations:** Instituto de Bioquímica Vegetal y Fotosíntesis (IBVF), CSIC-Universidad de Sevilla, Sevilla 41092, Spain; Instituto de Bioquímica Vegetal y Fotosíntesis (IBVF), CSIC-Universidad de Sevilla, Sevilla 41092, Spain; Departamento de Bioquímica Vegetal y Biología Molecular, Facultad de Biología, Universidad de Sevilla, Sevilla 41012, Spain; Instituto de Bioquímica Vegetal y Fotosíntesis (IBVF), CSIC-Universidad de Sevilla, Sevilla 41092, Spain; Departamento de Bioquímica Vegetal y Biología Molecular, Facultad de Biología, Universidad de Sevilla, Sevilla 41012, Spain; Instituto de Bioquímica Vegetal y Fotosíntesis (IBVF), CSIC-Universidad de Sevilla, Sevilla 41092, Spain; Departamento de Bioquímica Vegetal y Biología Molecular, Facultad de Biología, Universidad de Sevilla, Sevilla 41012, Spain; Instituto de Bioquímica Vegetal y Fotosíntesis (IBVF), CSIC-Universidad de Sevilla, Sevilla 41092, Spain

## Abstract

Carbon-flow-regulator A (CfrA) adapts carbon flux to nitrogen conditions in nondiazotrophic cyanobacteria. Under nitrogen deficiency, CfrA leads to the storage of excess carbon, which cannot combine with nitrogen, mainly as glycogen. *cfrA* overexpression from the arsenite-inducible, nitrogen-independent P*_arsB_* promoter allows analysis of the metabolic effects of CfrA accumulation. Considering that the main consequence of *cfrA* overexpression is glycogen accumulation, we examined carbon distribution in response to *cfrA* expression in *Synechocystis* sp. PCC 6803 strains impaired in synthesizing this polymer. We carried out a comparative phenotypic analysis to evaluate *cfrA* overexpression in the wild-type strain and in a mutant of ADP-glucose pyrophosphorylase (ΔglgC), which is unable to synthesize glycogen. The accumulation of CfrA in the wild-type background caused a photosynthetic readjustment although growth was not affected. However, in a ΔglgC strain, growth decreased depending on CfrA accumulation and photosynthesis was severely affected. An elemental analysis of the H, C, and N content of cells revealed that *cfrA* expression in the wild-type caused an increase in the C/N ratio, due to decreased nitrogen assimilation. Metabolomic study indicated that these cells store sucrose and glycosylglycerol, in addition to the previously described glycogen accumulation. However, cells deficient in glycogen synthesis accumulated large amounts of Calvin–Benson cycle intermediates as *cfrA* was expressed. These cells also showed increased levels of some amino acids, mainly alanine, serine, valine, isoleucine, and leucine. The findings suggest that by controlling *cfrA* expression, in different conditions and strains, we could change the distribution of fixed carbon, with potential biotechnological benefits.

## Teaser

Overexpression of the *carbon-flow-regulator A* gene in cyanobacteria strains unable to synthesize glycogen results in a massive accumulation of sugars.

## Introduction

Cyanobacteria, as photosynthetic organisms, have great potential for the sustainable production of different compounds of biotechnological interest (Tan et al. 2022; Toepel et al. 2023). The fundamental advantage of these organisms lies in their ability to transform CO_2_ into high-value products using solar energy. They present a high adaptive capacity based on their great metabolic plasticity (Xiong et al. 2017). An exhaustive characterization of cyanobacterial physiology and metabolism is key to the development of any biotechnological strategy. The model cyanobacterium *Synechocystis* sp. PCC 6803 (hereinafter *Synechocystis*) has been extensively studied and its global metabolism has been recently reviewed (Mills et al. 2020). In relation to carbon metabolism, recent studies have analyzed and quantified the contribution of different metabolic pathways under different conditions (Doello et al. 2018; Shinde et al. 2020; Lucius et al. 2021; Schulze et al. 2022; Lu et al. 2023). Glycogen is the main carbon storage compound in cyanobacteria. The metabolism of this polymer contributes to growth in diurnal cycles and survival under different stress conditions (Cano et al. 2018; Luan et al. 2019; Ortega-Martínez et al. 2022). Recently, glycogen metabolism has been identified as an essential element for optimal cyanobacterial growth in the rapid light–dark cycle of low-Earth orbit, which is relevant for space travel (Bishé et al. 2023). Numerous works have tried to alter the metabolism of glycogen in cyanobacteria, either by increasing or decreasing its synthesis, in order to redirect the fixed carbon toward certain pathways of interest (Davies et al. 2014; Li et al. 2014; Sun et al. 2018; Velmurugan and Incharoensakdi 2018; Mittermair et al. 2021; Sivaramakrishnan and Incharoensakdi 2022). These engineering strategies of glycogen metabolism and their physiological effects in cyanobacteria have been reviewed (Luan et al. 2019). On many occasions, the target selected for these manipulations is the glucose-1-phosphate adenylyltransferase (*glgC*) gene that encodes ADP-glucose pyrophosphorylase (AGP). This enzyme generates ADP-glucose from glucose-1-phosphate and ATP and is generally considered the rate-limiting step that holds control over the entire glycogen accumulation process (Gründel et al. 2012). Knockout mutants of *glgC* are completely unable to synthesize glycogen as well as other compounds that depend on ADP-glucose for their synthesis, such as the osmolyte glucosylglycerol (GG) (Klähn and Hagemann 2011). In *Synechocystis*, the *glgC* mutants grow photoautotrophically in continuous light in a very similar way to the wild-type (WT) strain. However, they show a pleiotropic phenotype in other culture conditions, with loss of viability in light–dark cycles and an anomalous response to nitrogen deficiency (Carrieri et al. 2012, 2017; Gründel et al. 2012). This response includes a metabolic carbon overflow, in which part of the fixed carbon is directed toward the synthesis and excretion of organic acids, mainly pyruvate and 2-oxoglutarate (2-OG). This excretion is affected by light intensity and can also be triggered when nitrogen is available. Under an excess energy input, it is considered an alternative energy dissipation mechanism when the glycogen buffer is not present (Cano et al. 2018).

Carbon-flow-regulator A (CfrA) is a regulatory protein, highly conserved in cyanobacteria, involved in the adaptation of carbon flow to nitrogen deficiency. Its expression increases very markedly in *Synechocystis* during nitrogen control A (NtcA)-dependent acclimation to nitrogen starvation (Giner-Lamia et al. 2017; Muro-Pastor et al. 2020). During this adaptive response (chlorosis), a large amount of carbon is stored as glycogen. CfrA, also called PII-interacting regulator of carbon metabolism (PirC), facilitates this accumulation by inhibiting 3-phosphoglycerate mutase (PGAM), an enzyme whose activity directs fixed carbon toward lower glycolysis. CfrA also interacts with the PII regulatory protein, mainly as a function of the binding of 2-OG to the latter, and thus affects the CfrA/PGAM interaction (Orthwein et al. 2021). The overexpression of *cfrA* triggers the accumulation of glycogen even in the presence of a nitrogen source and regardless of the presence of PII. The cells reach a pseudo-chlorotic state with low levels of pigments but without growth arrest. In the case of nitrogen depletion, *cfrA* overexpressing strains respond in a similar way to the WT strain, developing chlorosis; however, the readdition of nitrogen does not allow the process to be reversed and the concomitant consumption of accumulated glycogen that takes place in the WT strain. On the contrary, the strains lacking CfrA revert chlorosis in an accelerated manner compared to the WT strain. They present a greater flow of carbon toward the lower glycolysis and the TCA cycle for its combination with nitrogen and the synthesis of proteins (Muro-Pastor et al. 2020). Consistent with these results, CfrA-deficient strains have been metabolically engineered to maximize polyhydroxybutyrate production under nitrogen-deficient conditions. This polymer is synthesized naturally in *Synechocystis* from acetyl-CoA (Ac-CoA), and therefore, its synthesis is favored in the absence of CfrA (Koch et al. 2020; Orthwein et al. 2021). Given these results, the modulation of *cfrA* expression could have an important influence on the distribution of endogenous carbon. Taking into account that CfrA promotes the storage of carbon as glycogen, we wonder what would be the effect of the overexpression of *cfrA* in a mutant strain ΔglgC, unable to synthesize this polymer. As mentioned above, the *glgC* mutation causes a metabolic overflow with accumulation of pyruvate and 2-oxoglutarate. The inhibition of PGAM mediated by CfrA in combination with the *glgC* mutation could give rise to an unexplored carbon redistribution. We have undertaken a comparative analysis of the overexpression of *cfrA* in a WT or a ΔglgC genetic background. In a WT strain, the overexpression of *cfrA* leads to a massive accumulation of glycogen and other carbon compounds such as sucrose or GG, without removal of the nitrogen source from the medium. However, in a ΔglgC strain, the accumulation of CfrA has drastic negative effects on photosynthetic activity and growth. The carbon is reallocated to different carbohydrates and specific amino acids like alanine, serine, or leucine.

## Results

### *cfrA* overexpression severely affects the growth of ΔglgC mutants

**Figure 1.**
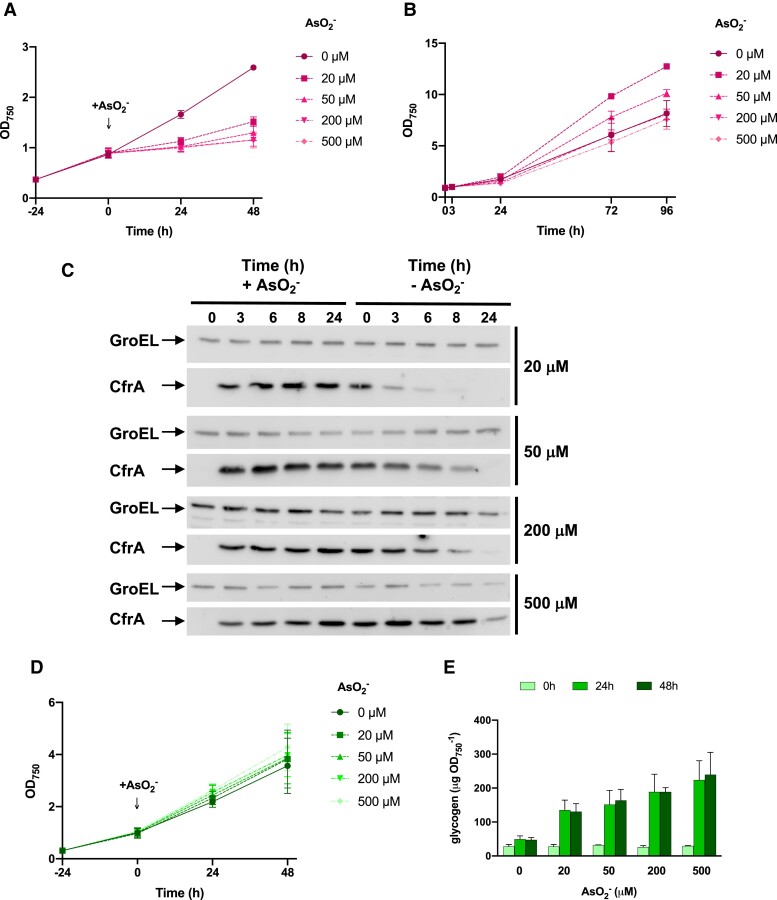
Analysis of the *cfrA* overexpressing strains Pars-cfrA and ΔglgC/Pars-cfrA. **A)** Growth analysis of ΔglgC/Pars-cfrA strain before and after the addition of the indicated amounts of arsenite. **B)** Growth of strain ΔglgC/Pars-cfrA after eliminating the arsenite present in the precultures at the indicated concentrations. **C)** Western-blot analysis of CfrA in ΔglgC/Pars-cfrA strain during the process of addition and subsequent removal of arsenite. Total protein crude extracts were obtained from cells corresponding to 2 OD_750_, resolved on SDS–PAGE, blotted and incubated with anti-CfrA antibodies. As a control for protein loading, membranes were incubated also with anti-GroEL antibodies. **D)** Growth analysis of Pars-cfrA strain before and after the addition of the indicated amounts of arsenite. **E)** Glycogen content of Pars-cfrA strain at 0, 24, and 48 h after arsenite addition at the indicated concentrations. Error bars in **A)**, **B)**, **D)**, and **E)** represent Sd of the mean values from 3 independent experiments.

To analyze the phenotype associated with *cfrA* overexpression in strains unable to synthesize glycogen, the previously described P*_arsB_*-controlled version of *cfrA* was introduced into a ΔglgC strain. In the resulting strain (ΔglgC/Pars-cfrA), the endogenous *cfrA locus* was eliminated as previously reported (Muro-Pastor et al. 2020). To rule out a deleterious effect of arsenite itself, the growth of the parental strains WT and ΔglgC was compared with those that overexpress *cfrA*. The addition of arsenite (500 *µ*M) only substantially affects the growth of the ΔglgC/Pars-cfrA strain (Supplementary Fig. S1). To further characterize the arsenite-dependent accumulation of CfrA and its effect on the ΔglgC/Pars-cfrA strain, it was first propagated in flasks using BG11C medium without arsenite, and then 150-ml cultures were inoculated under standard conditions in BG11C at 0.4 OD_750_ and supplemented with 1% (*v*/*v*) CO_2_. When these cultures reached ∼1 OD_750_, increasing amounts of arsenite were added to each culture (20, 50, 200, and 500 *µ*M). A control culture without arsenite was also included. As shown in Fig. 1A, the growth of the ΔglgC/Pars-cfrA strain was severely affected depending on the amount of arsenite added, with almost no growth at concentrations above 50 *µ*M. Considering the characteristics of the P*_arsB_* promoter, the transcription of *cfrA* gene should stop in the ΔglgC/Pars-cfrA strain by inducer removal. To analyze whether the cells were able to resume growth when the arsenite was removed, it was eliminated after 48 h of culture. The cells were washed and recultured without arsenite, setting the OD_750_ of all cultures to 1 at the beginning of the experiment. The OD_750_ and the CfrA amount in the cells were monitored. As shown in Fig. 1B, the cells recover growth depending on the amount of arsenite in the previous cultures, which determines the level of CfrA accumulation of the cells. Thus, cultures with lower concentrations of arsenite resumed growth earlier. CfrA accumulated soon after arsenite addition in the ΔglgC/Pars-cfrA strain, and it progressively disappeared from the cells when eliminating the arsenite in a way that depended on the concentration used in each culture (Fig. 1C). Cells from the control culture without arsenite showed slower growth in the reculture than would be expected since they did not accumulate CfrA. This behavior is most likely due to the fact that these cells have maintained active growth in the previous culture and are in the stationary phase at the beginning of the recultivation.

As previously described, the expression of *cfrA* by the addition of arsenite did not substantially affect the growth of the Pars-cfrA strain, observing only a slight increase in the OD_750_ as a function of the added arsenite (Fig. 1D) and a concentration-dependent accumulation of glycogen (Fig. 1E).

### *cfrA* overexpression in ΔglgC genetic background negatively affects the photosynthetic activity

**Figure 2.**
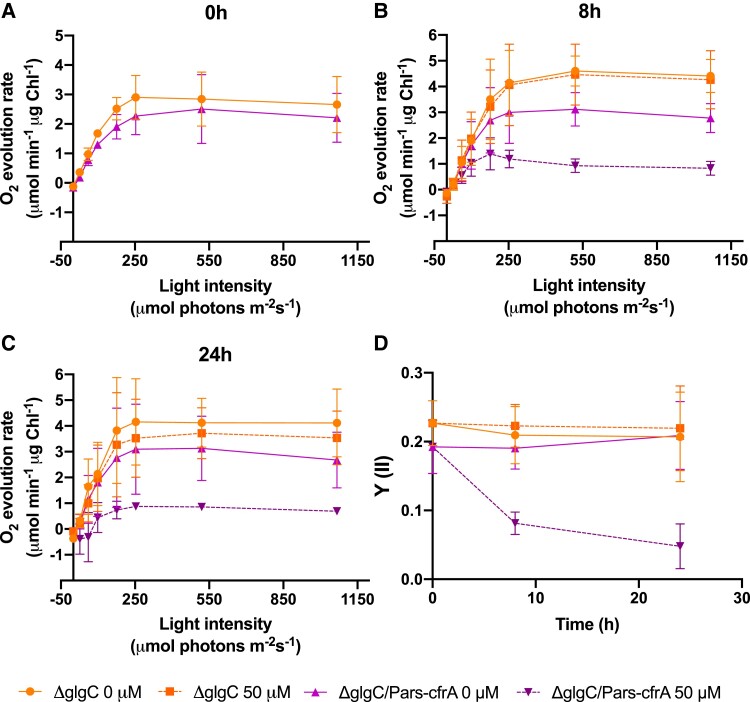
Effects of *cfrA* overexpression on photosynthetic parameters of ΔglgC/Pars-cfrA strain. **A** to **C)** Light saturation curves of cells from ΔglgC/Pars-cfrA strain and the control strain ΔglgC, normalized to chlorophyll content, measured before (0 h) or after (8 and 24 h) arsenite addition (50 *µ*M). **D)** PSII operating efficiency [Y(II)] of ΔglgC/Pars-cfrA and ΔglgC strains determined under growth light (60 *µ*mol photons m^−2^ s^−1^) before (0 h) or after (8 and 24 h) arsenite addition (50 *µ*Mm). Data are means ± Sd from 3 biological replicates in all cases.

**Figure 3.**
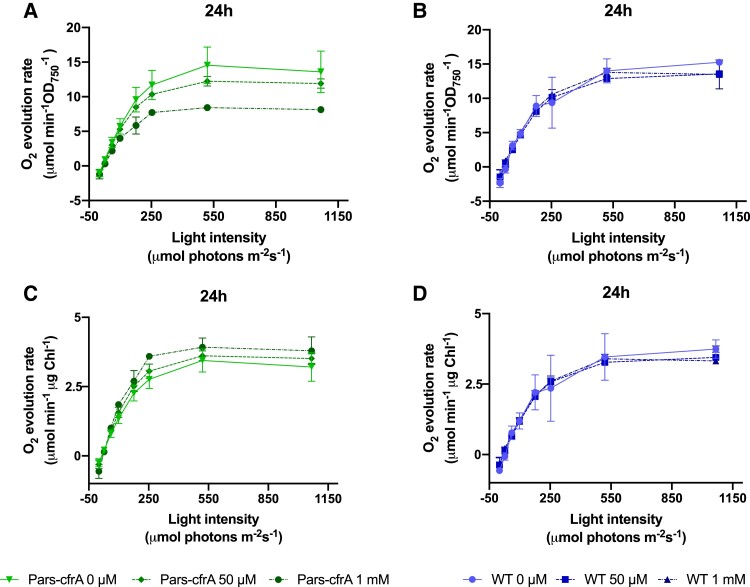
Effects of *cfrA* overexpression on photosynthetic parameters of Pars-cfrA strain. **A)** Light saturation curves of cells from Pars-cfrA strain equalized to OD_750_ of 1 at 24 h after arsenite addition (50 *µ*M or 1 mM). A control without arsenite was included. **B)** Light saturation curves of cells from WT strain in the same conditions. **C)** Light saturation curves of cells from Pars-cfrA strain 24 h after arsenite addition (50 *µ*M or 1 mM) normalized to chlorophyll content. **D)** Light saturation curves of cells from WT strain in the same conditions, normalized to chlorophyll content. Data are means ± Sd from 3 biological replicates in all cases.

The drastic effect that CfrA produces on the growth of the ΔglgC/Pars-cfrA strains led us to evaluate its photosynthetic capacity, from oxygen evolution rates, at different times after arsenite addition. As a control, the ΔglgC strain was analyzed under the same conditions. Oxygen-evolving rates at different light intensities were determined without inducer (0 h) and at 8 or 24 h after arsenite addition (50 *µ*M) (Fig. 2, A to C). After taking the initial samples, the culture of each strain was divided into 2, one was kept without the addition of inducer and the other was added arsenite. While before the addition of arsenite, the saturation curves were similar in the 2 strains analyzed (Fig. 2A), after the addition of the inducer, the light saturation curve of ΔglgC/Pars-cfrA strain reached a 3-fold lower maximum O_2_ evolution rate (Fig. 2, B and C). We also measured PSII operating efficiency [Y(II)] by pulse-amplitude-modulation fluorometry. Accordingly with the measurements of oxygen evolution, PSII efficiency strongly decreased in ΔglgC/Pars-cfrA strain after arsenite addition (Fig. 2D). Given that in a WT genetic background for glycogen synthesis (Pars-cfrA strain), the expression of *cfrA* does not affect growth, we decided to analyze the photosynthetic activity of this strain after the addition of arsenite. In previous works, we had observed that the addition of 1 mM of arsenite to this strain caused a significant decrease in photosynthetic pigments, chlorophyll, and phycocyanin (Muro-Pastor et al. 2020). Taking these data into account, the O_2_ evolution rate of this strain was analyzed 24 h after arsenite addition (50 *µ*M or 1 mM), using the WT strain as control. After the addition of arsenite, a decrease in the evolution of oxygen was observed in the Pars-cfrA strain, especially in the cultures treated with 1 mM of inducer (Fig. 3A). These treatments did not affect the oxygen evolution of the WT strain (Fig. 3B). However, when the oxygen evolution measurements were related to the amount of chlorophyll (Supplementary Fig. S2), both strains presented a similar rate, independent of the arsenite addition (Fig. 3, C and D), suggesting that the observed effect in the Pars-cfrA strain was due to a readjustment in the content of photosynthetic pigments.

### CfrA interacts with PGAM under nitrogen-replete conditions

The CfrA/PGAM interaction was demonstrated under nitrogen-deficient conditions, when *cfrA* is naturally expressed (Orthwein et al. 2021). We wanted to check here if this interaction also took place in our working conditions when *cfrA* was overexpressed in different genetic backgrounds under nitrogen-replete conditions. To this end, coimmunoprecipitation experiments were conducted. As PII protein is considered, the major CfrA interaction partner (Orthwein et al. 2021), a previously described PII lacking strain ΔglnB/Pars-cfrA was included, in order to promote the CfrA/PGAM interaction. All the phenotypic traits associated with the *cfrA* overexpression in nitrogen-replete conditions are maintained and even intensified in this strain (Muro-Pastor et al. 2020).

Cells from the different strains (Pars-cfrA, ΔglgC/Pars-cfrA, and ΔglnB/Pars-cfrA), cultivated in the presence of arsenite for 24 h, were used to obtain crude extracts. CfrA was immunoprecipitated using anti-CfrA antibodies coupled to protein A superparamagnetic beads. Controls with preimmune serum were carried out in parallel. Precipitated material was analyzed by LC-MS/MS using a TOF quadrupole and ProteinPilot v5.0.1 software for protein identification. A list of the first 30 proteins identified only with the immune serum and ordered by their score is available for each *cfrA* overexpressing strain. The score is based on the number of distinctive peptides identified for each protein and the false discovery rate (Supplementary Data Set S1). 2,3-Phosphoglycerate-independent PGAM coimmunoprecipitated with CfrA, only with the immune serum, in all the strains analyzed. This interaction was more robust in terms of score and number of peptides identified in the case of the strain lacking PII. These results clearly indicate that CfrA is acting through its interaction with PGAM in our working conditions.

### CfrA accumulation impact on intracellular element stoichiometry

**Figure 4.**
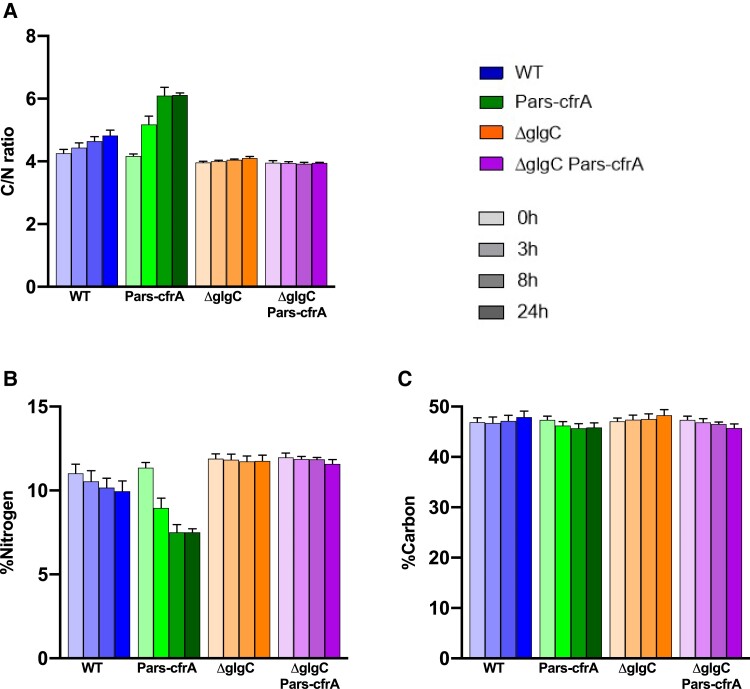
Chemical elemental analysis of the *cfrA* overexpressing strains Pars-cfrA and ΔglgC/Pars-cfrA, together with the control strains WT and ΔglgC. **A)** Elemental intracellular ratio of C/N, on a % dry mass basis, of the different strains analyzed before and after arsenite addition (100 *µ*M). **B)** Nitrogen content of the different strains analyzed before and after arsenite addition (100 *µ*M). **C)** Carbon content of the different strains analyzed before and after arsenite addition (100 *µ*M). Data are means ± Sd from 2 biological replicates with 3 technical replicates at each point.

**Figure 5.**
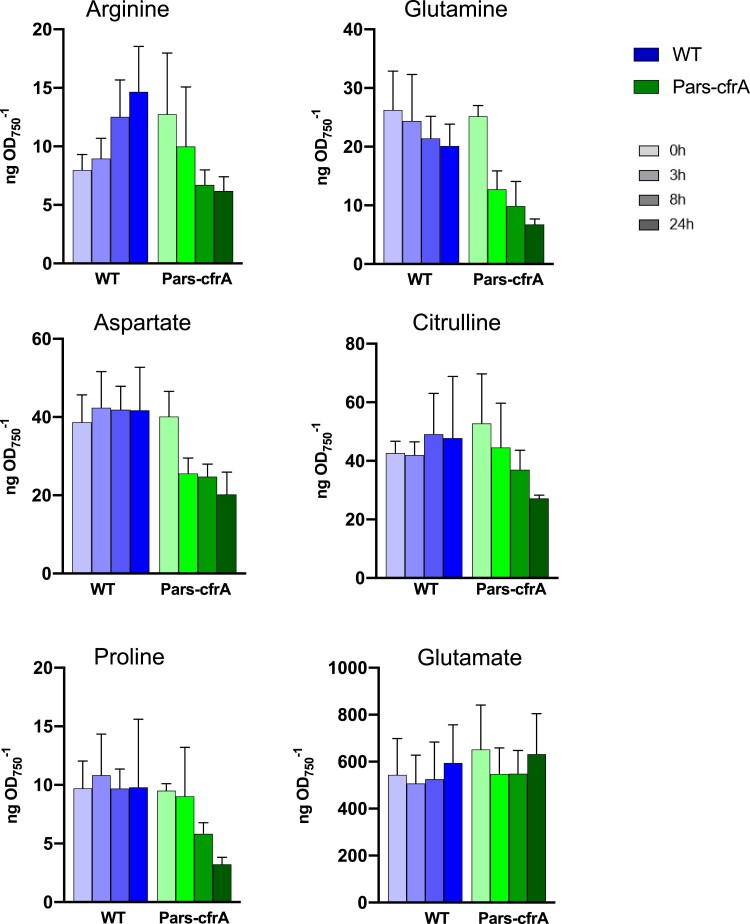
Time course LC-MS analysis of selected metabolites linked to the OAC cycle in the Pars-cfrA strain and the WT strain as a control. The quantification of these compounds over time (0, 3, 8, and 24 h) after the addition of arsenite (50 *µ*M) is shown. Each bar represents the metabolite level at a certain time, determined from 3 independent experiments with 3 technical replicates at each point. The error bars represent the Sd of the combined data. The values are in nanograms per OD_750_.

Taking into account the changes in the content of photosynthetic pigments and total protein associated with *cfrA* overexpression (Muro-Pastor et al. 2020), we set out to analyze the elemental composition of the biomass from Pars-cfrA and ΔglgC/Pars-cfrA strains as a function of CfrA accumulation. To eliminate potential interferences due to the addition of arsenite, the WT and ΔglgC strains were analyzed as controls. All the strains were grown under standard conditions, and *cfrA* expression was induced as in previously described experiments using 100 *µ*M arsenite. As shown in Fig. 4A, a slight and progressive increase in the C/N ratio (on a % dry mass basis) was observed in the WT strain after arsenite addition. This increase (13% at the end of the time analyzed) was most likely due to the age of the cultures, which leads to some storage of glycogen. A much more pronounced increase in the C/N ratio (24%) was observed for the strain Pars-cfrA already at 8 h after the addition of arsenite, reaching an increase of 53% 48 h after induction. However, in strains unable to synthesize glycogen, no substantial change in the C/N ratio was observed after the addition of arsenite. The observed effect on the C/N ratio is due in all cases to a decrease in nitrogen content rather than a change in total carbon (Fig. 4, B and C). These data agree with a clear decrease in nitrogen assimilation in the Pars-cfrA strain, as can be seen by the decrease in metabolites related to the GS-GOGAT cycle and the ornithine–ammonium cycle (OAC) such as glutamine, arginine, citrulline, aspartate, and proline. The glutamate did not present changes (Fig. 5).

### Metabolic analysis reveals clear differences in carbon partitioning associated with *cfrA* overexpression as a function of glycogen metabolism

**Figure 6.**
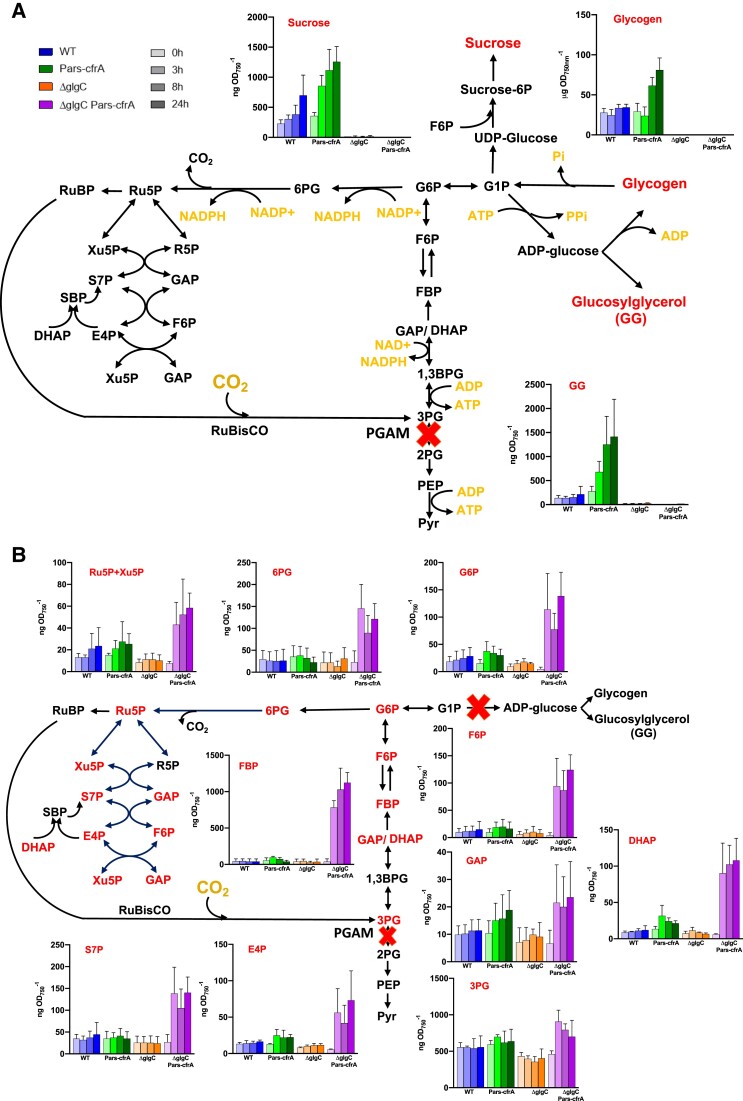
Time course LC-MS analysis of metabolites derived from the CO_2_ fixation product 3PG, as a function of *cfrA* expression. The PGAM reaction, negatively regulated by CfrA, is marked with a cross. **A)** Carbon storage compounds that accumulated substantially after *cfrA* induction in a WT background for ADP-glucose synthesis are marked in red. The quantification of these compounds over time (0, 3, 8, and 24 h) after the addition of arsenite (50 *µ*M) is shown. **B)** The reaction catalyzed by AGP, blocked in strains ΔglgC and ΔglgC/Pars-cfrA, is marked with a cross. The carbon compounds that strongly accumulated after the expression of *cfrA* in a strain blocked in the synthesis of ADP-glucose are marked in red. In **A)** and **B)**, each bar represents the metabolite level at a certain time, determined from 3 independent experiments with 3 technical replicates at each point. The error bars represent the Sd of the combined data. The values are in nanograms per optical density at 750 nm. The results of all analyzed carbohydrates are shown in Supplementary Fig. S3. 1,3BPG, 1,3-bisphosphoglycerate; 2PG, 2-phosphoglycerate; 3PG, 3-phosphoglycerate; 6PG, 6-phosphogluconate; DHAP, dihydroxyacetone phosphate; E4P, erythrose-4-phosphate; F6P, fructose-6-phosphate; FBP, fructose-1,6-bisphosphate; G1P, glucose-1-phosphate; G6P, glucose-6-phosphate; GAP, glyceraldehyde-3-phosphate; PEP, phosphoenolpyruvate; Pyr, pyruvate; R5P, ribose-5-phosphate; Ru5P, ribulose-5-phosphate; RuBP, ribulose-1,5-bisphosphate; S7P, sedoheptulose-7-phosphate; SBP, sedoheptulose-1,7-bisphosphate; Xu5P, xylulose-5-phosphate.

**Figure 7.**
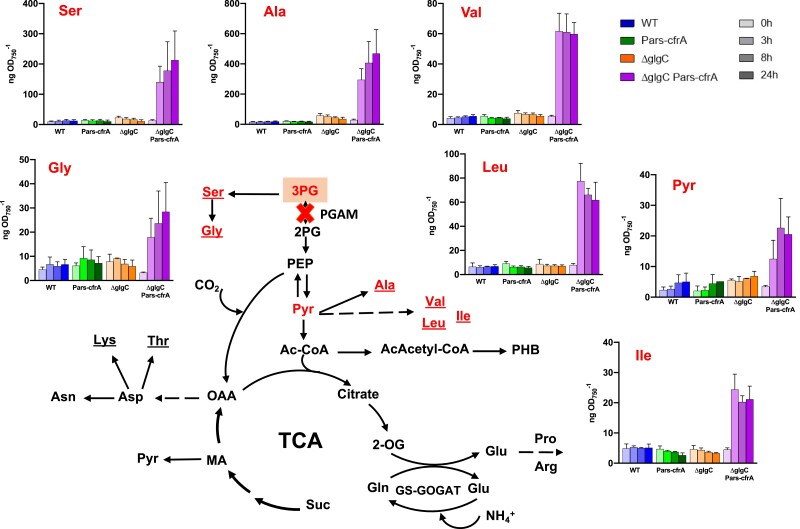
Time course LC-MS analysis of amino acids and pyruvate as a function of *cfrA* expression, depicted in a schematic representation of metabolic pathways from 3PG, including glycolysis, the TCA cycle, and the GS-GOGAT cycle. The PGAM reaction, negatively regulated by CfrA, is marked with a cross. Amino acids, derived from 3PG and pyruvate, that strongly accumulated after the expression of *cfrA*, in a strain blocked in the synthesis of ADP-glucose are marked in red. Each bar represents the metabolite level at a certain time, determined from 3 independent experiments with 3 technical replicates at each point. The error bars represent the Sd of the combined data. The results of all analyzed amino acids are shown in Supplementary Fig. S4 and other intermediaries in Supplementary Fig. S5. 2-OG, 2-oxoglutarate; 2PG, 2-phosphoglycerate; 3PG, 3-phosphoglycerate; AcAcetyl-CoA, aceto-acetyl-CoA; Ac-CoA, acetyl-CoA; MA, malate; OAA, oxaloacetate; PEP, phosphoenolpyruvate; PHB, polyhydroxybutyrate; Pyr, pyruvate: Suc, succinate.

In order to analyze the metabolic effects of *cfrA* overexpression in the different genetic backgrounds, we carried out a comparative global metabolomic study between the Pars-cfrA and ΔglgC/Pars-cfrA strains in a time course after the addition of arsenite (50 *µ*M). WT and ΔglgC strains were used as controls under the same conditions. Samples for the metabolic analysis were taken after 0, 3, 8, and 24 h from the induction of *cfrA* with arsenite. In a WT genetic background for glycogen synthesis, *cfrA* overexpression caused the accumulation of carbon storage compounds such as sucrose or GG, in addition to the already described glycogen production (Fig. 6A). However, when *cfrA* was induced in a ΔglgC strain, virtually all carbon compounds upstream of the 3PG experienced a substantial increase early with the addition of the inductor (Fig. 6B). The accumulation of all these sugars phosphate (hexoses, pentoses, trioses, etc.) took place rapidly, reaching high levels in almost all cases already 3 h after the addition of the *cfrA* inducer. This is consistent with the rapid accumulation observed for CfrA protein (Fig. 1C). Especially important was the rise in fructose-1,6-bisphosphate, more than 20 times, 3 h after the addition of arsenite, and 30 times higher after 24 h of induction in ΔglgC/Pars-cfrA strain. In a similar range, sedoheptulose-7-phosphate, erythrose-4-phosphate (E4P), fructose-6-phosphate, glucose-6-phosphate, dihydroxyacetone phosphate, and 6-phosphogluconate also increased at 3 and 24 h of the induction. None of these compounds accumulated appreciably in the Pars-cfrA strain compared to the control WT strain after adding arsenite. Since the photosynthetic assimilation of carbon is a main source of intermediates for amino acid synthesis, we also analyzed amino acid levels as a function of *cfrA* expression. In the ΔglgC/Pars-cfrA strain, unable to synthesize glycogen, the induction of *cfrA* led to a large increase in some amino acids (Fig. 7). Thus, serine and glycine, whose synthesis derives from 3PG, increased 15 and 9 times, respectively, after *cfrA* induction. It is interesting to note that 3PG could be accumulated by the action of CfrA on the PGAM enzyme and that in fact increased in the ΔglgC/Pars-cfrA strain after the addition of arsenite (Fig. 6B). Nonetheless, the other amino acids that increased substantially in this strain: alanine (16 times), whose synthesis takes place directly from pyruvate by the action of alanine dehydrogenase, valine (10 times), leucine (8 times), and isoleucine (5 times) originate from pyruvate, a metabolite that is found downstream of the action of PGAM in the glycolytic pathway. None of these amino acids undergo appreciable changes after the addition of arsenite in the Pars-cfrA strain (Fig. 7). The full spectra of the metabolites analyzed by LC-MS are shown in Supplementary Figs. S3 to S6. An increase of around 2-fold was observed in the pool of tryptophan and tyrosine of strain ΔglgC/Pars-cfrA after the addition of arsenite (Supplementary Fig. S4), which is in agreement with the increase in E4P, a precursor of their synthesis (Fig. 6B). It is worth noting that glutamate, the more abundant amino acid in *Synechocystis*, exhibited low fluctuation in the analyzed conditions. As expected, the ΔglgC strains do not accumulate ADP-glucose; however, there are no substantial differences in the accumulation of UDP-glucose between the different genetic backgrounds analyzed (Supplementary Fig. S6).

## Discussion

Here, we describe the metabolic effects of *cfrA* overexpression in different strains, based on their ability to synthesize ADP-glucose and its derivatives, mainly glycogen. The results show that the overexpression of *cfrA* in cells unable to synthesize ADP-glucose, and therefore glycogen, severely slows down growth, even stopping it at high concentration of the inducer arsenite (0.5 mM) (Fig. 1A; Supplementary Fig. S1). However, the expression of *cfrA* in WT cells for the synthesis of reserve carbohydrates does not cause negative effects on growth (Fig. 1D; Supplementary Fig. S1). The fact that the removal of the inducer arsenite and the progressive decrease of CfrA reverse the negative effect on the growth of the ΔglgC/Pars-cfrA strain clearly indicates that the accumulation of CfrA is the cause of this effect, also demonstrating that it is a reversible process that does not permanently compromise cell viability, at least at the times tested (Fig. 1B).

The photosynthetic analysis carried out shows a clearly different effect of the overexpression of *cfrA* in the 2 genetic backgrounds analyzed. In the ΔglgC/Pars-cfrA strain, a double blockage occurs in the flow of carbon, on the one hand toward the synthesis of reserve compounds (ΔglgC mutation) and on the other toward the synthesis of biomass by combining carbon with nitrogen (mediated by CfrA/PGAM interaction). This blockage leads to a massive accumulation of Calvin–Benson cycle intermediates (Fig. 6B), which is probably the cause of the drastic decrease in photosynthetic activity (Fig. 2). However, in the Pars-cfrA strain, a decrease in cellular chlorophyll content is observed, especially if a high concentration of inducer is added (Supplementary Fig. S2). This leads to a logical decrease in the oxygen evolution rate per cell (Fig. 3A), but this decrease was not observed when this rate was relativized by the chlorophyll content (Fig. 3C). These results are consistent with the previously described decrease in photosynthetic pigments of the Pars-cfrA strain upon induction of *cfrA* expression (Muro-Pastor et al. 2020) and highlight the metabolic readjustment mediated by this protein when it is expressed under sufficient nitrogen conditions.

Carbon storage in the form of glycogen tends to accumulate in bacteria at stationary phase, when biomass synthesis is lower and less carbon is combined with nitrogen. This is probably the cause of the progressive increase of the C/N ratio during growth in the WT strain and the concomitant decrease of nitrogen that we have observed in the elemental analysis (Fig. 4). These variations do not occur in strains unable to synthesize glycogen (ΔglgC background). Interestingly, the Pars-cfrA strain presents a much more substantial decrease in the amount of nitrogen and therefore an increase in the C/N ratio, in agreement with the previously observed low GS activity and protein content in this strain (Muro-Pastor et al. 2020). The decrease in the amino acids glutamine, proline, arginine, and aspartic acid as well as citrulline, intermediate of the synthesis of arginine, (Fig. 5), suggests a decrease in the assimilation of ammonium as well as in the functionality of the OAC cycle, the key storage and remobilization center for cyanobacterial nitrogen (Zhang et al. 2018). These data clearly confirm the role of CfrA in the regulation of carbon flux, favoring its accumulation in glycogen and to the detriment of its combination with nitrogen for the synthesis of amino acids and proteins.

In addition to the accumulation of carbon in the form of glycogen, the Pars-cfrA strain accumulates substantial amounts of sucrose and GG, compared to the WT strain, in response to the addition of arsenite. These sugars are synthesized in *Synechocystis* and other cyanobacteria as osmoprotectants in response to salt stress (Klähn and Hagemann 2011). The amount of GG accumulated in the Pars-cfrA strain under our conditions (absence of salt stress) represents approximately 10% of the amount accumulated in the WT strain in the presence of 0.5 M NaCl (Díaz-Troya et al. 2020). These results indicate that the synthesis of sucrose or GG represents an additional carbon sink to the accumulation of glycogen in response to the action of CfrA. In the case of the ΔglgC/Pars-cfrA strain, the GG synthesis does not take place since it requires ADP-glucose. No accumulation of sucrose was observed in this strain either, under the conditions of this work, despite the fact that sucrose is synthesized from UDP-glucose and the quantity of this compound is not affected in the ΔglgC mutants (Supplementary Fig. S6).

In this work, the metabolomic analysis has been carried out using a reduced concentration of arsenite (50 *μ*M) for the induction of *cfrA* expression. This concentration makes it possible to observe the phenotype associated with *cfrA* overexpression in a WT genetic background for glycogen synthesis (strain Pars-cfrA), previously described using 1 mM arsenite (Muro-Pastor et al. 2020), avoiding on the other hand some transient metabolic effects observed at this concentration. On the other hand, given the drastic effects of CfrA accumulation in the ΔglgC/Pars-cfrA strain, the use of a limited amount of arsenite guarantees sublethal conditions, at least for short periods of time.

As a whole, the metabolic data obtained clearly indicate very different consequences of *cfrA* overexpression if the strains are capable or not of synthesizing ADP-glucose and its derivatives. Under the conditions studied, there is no relevant increase in levels of any intermediate derived from 3PG or amino acid in the Pars-cfrA strain (Figs. 6B and 7; Supplementary Figs. S3 to S5), with respect to the WT strain, indicating that the CfrA-mediated blockage shifts carbon primarily toward the aforementioned glycogen, sucrose, and GG sinks. It is worth noting that under these conditions (50 *μ*M arsenite), a minimal photosynthetic readjustment occurs in this strain (Fig. 3 and Supplementary Fig. S2). By contrast, in the ΔglgC/Pars-cfrA strain, there is an enormous accumulation of sugars and a drastic decrease in photosynthetic activity, stopping growth while the expression of *cfrA* is maintained by the presence of the inducer. Previous studies with ΔglgC mutants have revealed growth delays in high lighting conditions indicating that energy management and the balance between photosynthate and its utilization is impaired in these mutants. In fact, the redistribution of carbon toward the production of a heterologous compound partially rescues these effects. The accumulation of carbohydrates that takes place in these strains could have negative feedback effects on photosynthetic activity (Miao et al. 2003; Li et al. 2014). On the other hand, it has been shown that the availability of carbon sinks conditions photosynthetic performance (Holland et al. 2016). Considering this, it is not surprising to observe that the expression of *cfrA*, which negatively affects carbon flux toward glycolysis and biomass synthesis, causes drastic negative effects in a ΔglgC genetic background where the glycogen sink does not operate. It is striking the enormous accumulation of fructose-1,6-bisphosphate in the ΔglgC/Pars-cfrA strain. This metabolite, substrate of fructose-1,6/sedoheptulose-1,7-bisphosphatase (F/SBPase), is found around 9 times more abundant than the product of the reaction of this enzyme, fructose-6-phosphate. This would point to a possible inhibition of F/SBPase activity after the expression of *cfrA* in a ΔglgC background. The other substrate of this enzyme, sedoheptulose-1,7-bisphosphate, could not be quantified in this work as no standard was available. Interestingly, recent research on the metabolic regulation of the Calvin–Benson cycle in *Synechocystis* has revealed the importance of this type of regulation for flow control in this cycle and specifically acting on F/SBPase. These studies have demonstrated in vitro inhibition of F/SBPase under oxidizing conditions mediated by glyceraldehyde-3-phosphate (GAP) (Sporre et al. 2023). Our results showed a clear increase in GAP in ΔglgC/Pars-cfrA strain associated with *cfrA* expression (Fig. 6B). In addition, the conditions could be oxidizing given the drastic decrease in photosynthetic activity (Fig. 4). Therefore, a GAP-mediated inhibition of F/SBPase could take place under these conditions.

*cfrA* overexpression causes an increase in the concentration of 2-OG in both WT and ΔglgC genetic backgrounds (Supplementary Fig. S5). 2-OG is a corepressor of the NdhR regulator that acts on the genes of the Ci transport systems (Forchhammer and Selim 2020). Thus, 2-OG increase could negatively affect the activity of the Calvin–Benson cycle and signal a situation of carbon excess and a limitation in the assimilation of nitrogen (low GS-GOGAT activity) that is associated with the accumulation of CfrA (Muro-Pastor et al. 2020). This excess of carbon that cannot be channeled to glycogen in the ΔglgC/Pars-cfrA strain leads to the already mentioned accumulation of sugars and also of amino acids with a high carbon content. Interestingly, the synthesis of some of these amino acids that accumulate in large quantities in the ΔglgC/Pars-cfrA strain takes place from pyruvate, a metabolite that also accumulates in this strain after arsenite addition (Fig. 7). Since pyruvate is found in the glycolytic pathway downstream of the CfrA-mediated point of the regulation (PGAM), several questions arise. The data indicate that probably in our conditions of *cfrA* overexpression, the PGAM activity is not completely blocked. Furthermore, it is possible that PGAM isoenzymes insensitive to CfrA operate to some extent under these conditions. In this sense, several PGAM isoenzymes are annotated in *Synechococcus elongatus* PCC 7942 and homologous sequences exist in *Synechocystis* (Jablonsky et al. 2013; Broddrick et al. 2016). On the other hand, the aforementioned overflow of the ΔglgC strains would favor the accumulation of carbon skeletons (2-OG, pyruvate, Ac-CoA, malate) (Supplementary Fig. S5). Alternatively, some of these compounds could come from other operative pathways in cyanobacteria that bypass PGAM activity, such as the Entner–Doudoroff pathway or phosphoketolase. Specifically, the accumulation of Ac-CoA observed when overexpressing *cfrA* in both WT and ΔglgC genetic backgrounds could be an indicative of phosphoketolase function (Xiong et al. 2015, 2017; Chen et al. 2016; Bachhar and Jablonsky 2020). All these compounds, in the presence of a nitrogen source (always present in our cultures), would give rise to the synthesis of certain amino acids.

From a biotechnology standpoint, the present metabolic characterization of strains that overexpress the *cfrA* regulator in different genetic backgrounds reveals their high potential for carbon channeling to compounds of interest. Numerous studies have attempted to suppress glycogen synthesis for this purpose. However, contrary to what was initially expected, this limits rather than increases production in some cases (Qiao et al. 2018; Cantrell et al. 2023). These data point to the utility of our Pars-cfrA strain that can store large amounts of glycogen without the need to eliminate the nitrogen source or compromise growth. On the other hand, the glycogen-rich biomass of this strain could be used in coupled cultures to supply a carbon source to a heterotrophic organism that would produce a compound of interest, a strategy already analyzed with other cyanobacteria (Comer et al. 2020). Similarly, in the case of strain ΔglgC/Pars-cfrA, the double blockage toward the synthesis of glycogen and biomass could favor the carbon sink for the synthesis of other compounds of interest and could release the negative effect of *cfrA* overexpression in this strain.

## Materials and methods

### Culture conditions

*Synechocystis* sp. PCC 6803 derivative strains were grown photoautotrophically at 30°C on BG11 medium (Rippka 1988), supplemented with 1 g L^−1^ NaHCO_3_ (BG11C) and bubbled with 1% (*v*/*v*) CO_2_ in air, under continuous illumination (50 to 70 *µ*mol of photons m^−2^ s^−1^; 4,000K LED lights), hereafter standard conditions. For plate cultures, 1% (*w*/*v*) Bacto agar (Difco) and the required antibiotics were added (50 *µ*g ml^−1^ kanamycin, 2.5 *µ*g ml^−1^ spectinomycin, 2.5 *µ*g ml^−1^ streptomycin, and 20 *µ*g ml^−1^ chloramphenicol). Sodium arsenite (NaAsO_2_) at different concentrations was added when required. Growth was monitored via optical density at 750 nm (OD_750_). Chlorophyll concentration was determined in methanolic extracts (Mackinney 1941).

### Generation of mutant strains

To generate the ΔglgC/Pars-cfrA strain, the previously described parsBnrsΔcfrA and p*Δ*cfrA plasmids (Muro-Pastor et al. 2020) were used to transform the ΔglgC strain. *Synechocystis* strains used in this work are stated in Table 1.

**Table 1.** *Synechocystis* strains used in this work

Name	Description	Reference
**WT**	*Synechocystis* sp. PCC 6803 WT	
**Pars-cfrA**	Δ*cfrA::aadA*^+^, *nrsD*::P*_arsB_*-*cfrA:npt*, Sm/Sp^r^, Km^r^	Muro-Pastor et al. (2020)
**ΔglnB/Pars-cfrA**	Δ*glnB::cm, nrsD*::P*_arsB_*-*cfrA:npt*, Cm^r^, Km^r^	Muro-Pastor et al. (2020)
**ΔglgC**	Δ*glgC::cm*, Cm^r^	Díaz-Troya et al. (2014)
**ΔglgC/Pars-cfrA**	Δ*glgC::cm*, Δ*cfrA::aadA*^+^, *nrsD*::P*_arsB_*-*cfrA:npt*, Cm^r^, Sm/Sp^r^, Km^r^	This work

### Preparation of crude extracts and western blot analysis

For the analysis of protein abundance, 2 U OD_750_ were harvested and resuspended in 100 *µ*l 50 mM HEPES-NaOH buffer (pH 7.0), KCl 50 mM, and PMSF 1 mM. The cOmplete ULTRA tablets of Protease Inhibitor Cocktail from ROCHE were used. Crude extracts were prepared by mechanical disruption using glass beads by 10 cycles of 1-min vortexing/resting on ice. After centrifugation (15,000 × *g* 20 min at 4°C), the soluble fraction was recovered. For western blot analysis, proteins were fractionated on 12% (*w*/*v*) SDS–PAGE (Laemmli 1970) and transferred to nitrocellulose membranes (Bio-Rad). Blots were blocked with 5% (*w*/*v*) nonfat dry milk (AppliChem) in PBS-Tween 20. Antisera were used at the following dilutions: Anti-CfrA (Muro-Pastor et al. 2020) (1:10,000) and anti-*Escherichia coli* GroEL (1:45,000, Sigma-Aldrich). The ECL Prime Western Blotting Detection Reagent (Amersham, Cytiva) was used to detect the different antigens with anti-rabbit secondary antibodies (1:25,000) (Sigma-Aldrich).

### Photosynthetic measurements

Oxygen evolution was determined on cell cultures using a Clark-type electrode Chlorolab 2+ System (Hansatech) at 30°C with continuous stirring. Cells were harvested at different times and adjusted to OD_750_ = 1. To prevent carbon limitation, cultures were supplemented with 10 mM NaHCO_3_ just before measurements. For O_2_ evolution at light saturation curves, light was provided by an LED light source (LED1, Hansatech). Chlorophyll fluorescence measurements were performed with a DUAL-PAM-100 (Walz) using intact cells at room temperature. Before measurements, cell suspensions (OD_750_ = 1) were dark adapted for 10 min. The effective quantum yield of PSII, Y(II), was calculated.

### Glycogen content determination

Glycogen was determined as previously described (Gründel et al. 2012) with some modifications. 2 U OD_750_ were harvested and resuspended in 30% (*w*/*v*) KOH, mixed by vortexing and incubated for 2 h at 95°C. Glycogen was then precipitated by adding cold ethanol and incubating overnight at −20°C. Glycogen granules were harvested by centrifugation (15,000 × *g* for 10 min at 4°C) and washed with cold ethanol to eliminate KOH traces. Pellets were resuspended in 100 mM sodium acetate (pH 5.2) and enzymatically hydrolyzed to glucose with 10 U amyloglucosidase (from *Aspergillus niger*, Sigma) at 55°C overnight. A calibration curve using commercial glycogen was also prepared. Released glucose was determined in all samples by the glucose oxidase/peroxidase method (Sigma GAGO-20) for 30 min at 30°C. The reaction was stopped by adding H_2_SO_4_ to a final concentration of 4.8 N, and absorbance was measured at 540 nm on a Varioskan multiplate reader.

### Immunoprecipitation assays

Superparamagnetic beads with recombinant Protein A (Dynabeads Protein A from Invitrogen) were washed twice with HEPES 50 mM pH 7 buffer using a magnetic holder for microtubes. Two volumes of immune or preimmune serum (100 *µ*l) were added to the beads suspension, and the tubes were incubated at 4°C for 2 h with rotation. Beads were washed with HEPES 50 mM pH 7 buffer and antibodies were cross-linked to beads using BS^3^ (Thermo Scientific) following the instructions of the supplier.

Crude cell extracts were obtained from the different *cfrA* overexpressing strains after 24 h of treatment with arsenite using glass beads in MES buffer (25 mM Mes pH 6.5, 5 mM CaCl_2_, 10 mM MgCl_2_, and 20% [*v*/*v*] glycerol) supplemented with Complete Protease Inhibitor Cocktail EDTA-free (Roche). Soluble proteins were recovered after a 25-min centrifugation at 20,000 × *g*. A volume of crude extract equivalent to 100 OD_750_ was used for each immunoprecipitation assay. Crude extracts were incubated with antibody-cross-linked Dynabeads overnight at 4°C with rotation. Dynabeads were then washed twice with the same buffer, and the precipitated material was eluted with standard SDS protein buffer without ß-mercaptoethanol and heated at 50°C for 10 min.

### LC-MS/MS protein identification

Protein samples were precipitated with a TCA:acetone mix. The protein precipitate was resuspended with a 0.2% (*w*/*v*) RapiGest solution (Waters) in 50 mM ammonium bicarbonate; 5 mM DTT was added and incubated for 30 min at 60°C. Then, iodoacetamide (IAA) was added to a final concentration of 10 mM by incubating 30 min in the dark at room temperature. Trypsin digestion was done at 37°C overnight. Trypsin was quenched with formic acid and samples were injected into the liquid chromatography equipment with tandem MS (LC-MS/MS).

The analysis was performed on a triple TOF quadrupole (5,600 plus from Sciex) equipped with a nanoelectrospray source, coupled to a nano-HPLC (Eksigent). The software used for the control of the equipment, as well as for the acquisition and processing of data, was Analyst TF 1.7. The peptides were first loaded into a trap column (Acclaim PepMap 100 C18, 5 *µ*m, 100 Å, 100 *µ*m id × 20 mm, Thermo Fisher Scientific) isocratically in 0.1% (*v*/*v*) formic acid/5% (*v*/*v*) acetonitrile at a flow of 3 *µ*l min^−1^ for 10 min. Subsequently, they were eluted in a reverse phase analytical column with the “emitter” already incorporated (New Objective PicoFrit column, 75 *µ*m id × 250 mm, packed with Reprosil-PUR 3 *µ*m), using a linear gradient of 5% to 35% of solvent B for 60 min at a flow of 250 nl min^−1^. Solvent A was 0.1% formic acid (*v*/*v*) and B, acetonitrile with 0.1% formic (*v*/*v*). A standard (digest of *E. coli* beta-galactosidase) was used to self-calibrate and control the equipment’s sensitivity and chromatographic conditions.

The source voltage was selected at 2,600 V and the heater temperature was maintained at 100°C. Gas 1 was used at 15 psi, Gas 2 at 0, and curtain gas at 25 psi. For protein identification experiments, the acquisition was carried out with a data-dependent acquisition method, consisting of a TOF-MS with a sweep window of 400 to 1,250 *m*/*z*, acquisition time of 250 ms, followed by 50 MS/MS with a sweep window of 230 to 1,500 *m*/*z*, acquisition time of 65 ms, and a cycle time of 3.54 s.

For protein identification, ProteinPilot v5.0.1 software (Sciex) was used, with a Paragon method, with trypsin as enzyme and IAA as alkylating agent of cysteines, using as protein database, the reference proteome of *Synechocystis* in FASTA format from Uniprot.org merged with the Sciex database of contaminants. Rabbit or human contaminants were removed, as were *Synechocystis* ribosomal proteins. Proteins identified with both the preimmune and immune serum were equally eliminated. Protein identification was carried out in the protein analysis service of the Instituto de Bioquímica Vegetal y Fotosíntesis (IBVF).

### Chemical elemental analysis

The elemental analysis was carried out in the microanalysis service of the Centro de Investigación Tecnológica e Innovación Universidad de Sevilla, using a LECO elemental analyzer, model TRUSPEC CHNS MICRO. One to 2 mg of lyophilized cells were subjected to complete and immediate combustion with pure oxygen at a maximum temperature of 1050°C. The different combustion products (CO_2_, H_2_O, and N_2_) were subsequently quantified using an infrared cell or a thermal conductivity cell.

### Quantification of intracellular metabolites

For intracellular metabolite quantification, 1 to 3 U OD_750_ were quickly collected by centrifugation (15,000 × *g* for 30 s at 4°C) and immediately frozen in liquid N_2_ for storage at −80°C until use. Metabolite extraction from frozen pellets was carried out as previously described (Ortega-Martínez et al. 2022). For LC-MS analysis, chromatographic separation was performed with an XSELECT HSS XP 150 mm × 2.5 *µ*m column (Waters) in an Exion HPLC (Sciex) connected to a Qtrap 6500 (Sciex) operating in negative mode. In the case of amino acid analysis, a Kinetex (Phenomenex) XB-C18 100 Å, 100 × 4.6 mm column was used. This column was connected to the same Exion HPLC but operating in positive mode. Sample data were acquired and processed with Analyst and SciexOs software. For quantification of the total amount of the metabolites, different known concentrations of each standard were used. The quantification of metabolites was carried out in the chromatography service of the IBVF.

### Accession numbers

The accession numbers of the proteins identified in the immunoprecipitation assays are indicated in Supplementary Data Set S1.

## Supplementary Material

kiae083_Supplementary_Data

## Data Availability

The data underlying this article are available in the article and in its online supplementary material.

## Supplementary data

The following materials are available in the online version of this article.

**Supplementary Figure S1.** Effect of *cfrA* expression on the growth of the strains studied.

**Supplementary Figure S2.** Effect of *cfrA* expression on the chlorophyll content of Pars-cfrA strain.

**Supplementary Figure S3.** Time course LC-MS analysis of all analyzed carbohydrates as a function of *cfrA* expression.

**Supplementary Figure S4.** Time course LC-MS analysis of all analyzed amino acids as a function of *cfrA* expression.

**Supplementary Figure S5.** Time course LC-MS analysis of some metabolic intermediaries analyzed as a function of *cfrA* expression.

**Supplementary Figure S6.** Time course LC-MS analysis of ADP-glucose (ADP-Glc) and UDP-glucose (UDP-Glc) analyzed as a function of *cfrA* expression.

**Supplementary Data Set S1.** List of proteins identified by LC-MS/MS in immunoprecipitation assays.

## Dive Curated Terms

The following phenotypic, genotypic, and functional terms are of significance to the work described in this paper:

HEPES CHEBI: CHEBI:46756

glycogen CHEBI: CHEBI:28087

PHB Gramene: AT2G34710
PHB Araport: AT2G34710
