# Supplementary material for: Double blocking of carbon metabolism causes a large increase of Calvin–Benson cycle compounds in cyanobacteria
Source: Plant Physiol. 2024 Feb 20;195(2):1491–505. doi: 10.1093/plphys/kiae083 (PMC11142378; doi:10.1093/plphys/kiae083)
Supplement: kiae083_Supplementary_Data [file kiae083_supplementary_data.zip › Supplemental Figures S1S6.pdf]

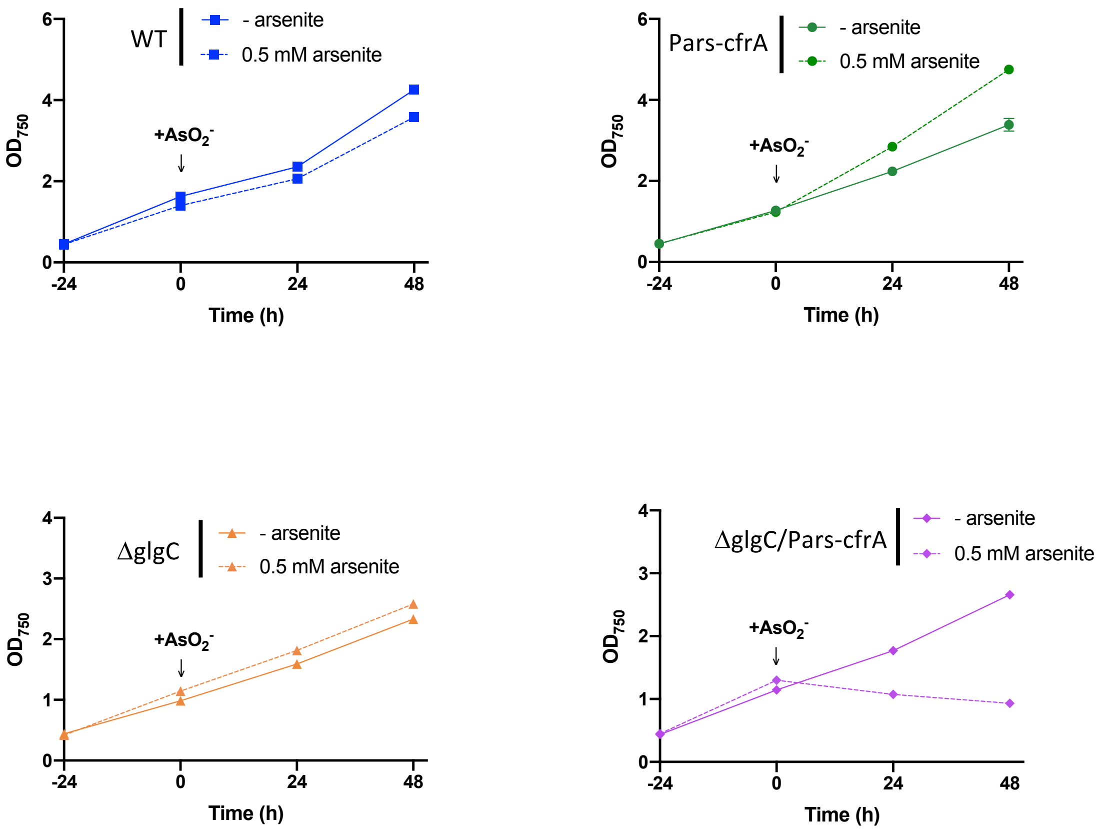

Supplemental Figure S1. Effect of *cfrA* expression on the growth of the strains studied. Growth analysis of Pars-cfrA and ΔglgC/Pars-cfrA strains, together with the parental strains WT and ΔglgC, respectively, before and after the addition of arsenite (0.5 mM). The data shown correspond to a representative experiment of the effect of arsenite in the different strains.

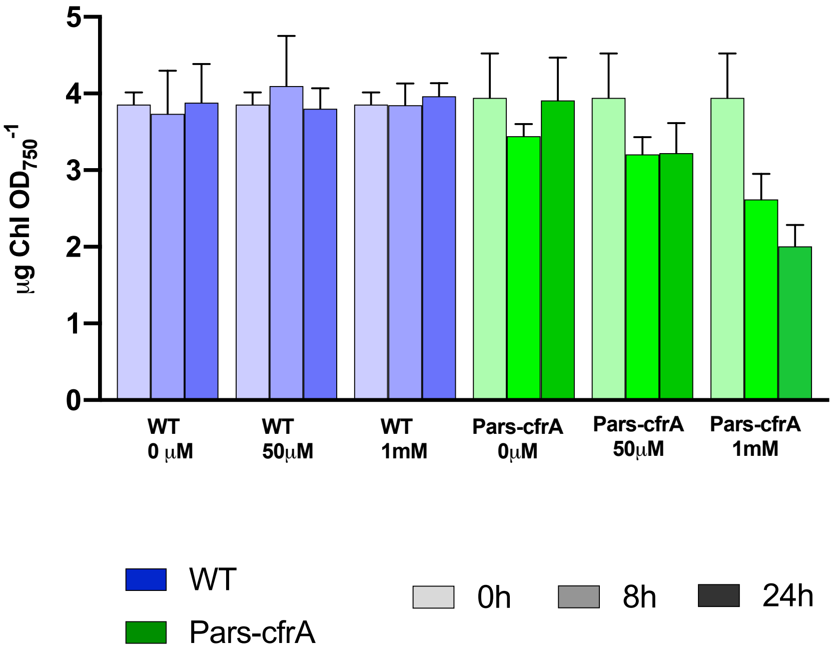

Supplemental Figure S2. Effect of *cfrA* expression on the chlorophyll content of Pars-cfrA strain. Chlorophyll content, relative to the OD_{750}, of the different strains before and after (0, 8, 24 h) the addition of arsenite at the indicated concentrations. Data are means ± SD from three biological replicates in all cases.

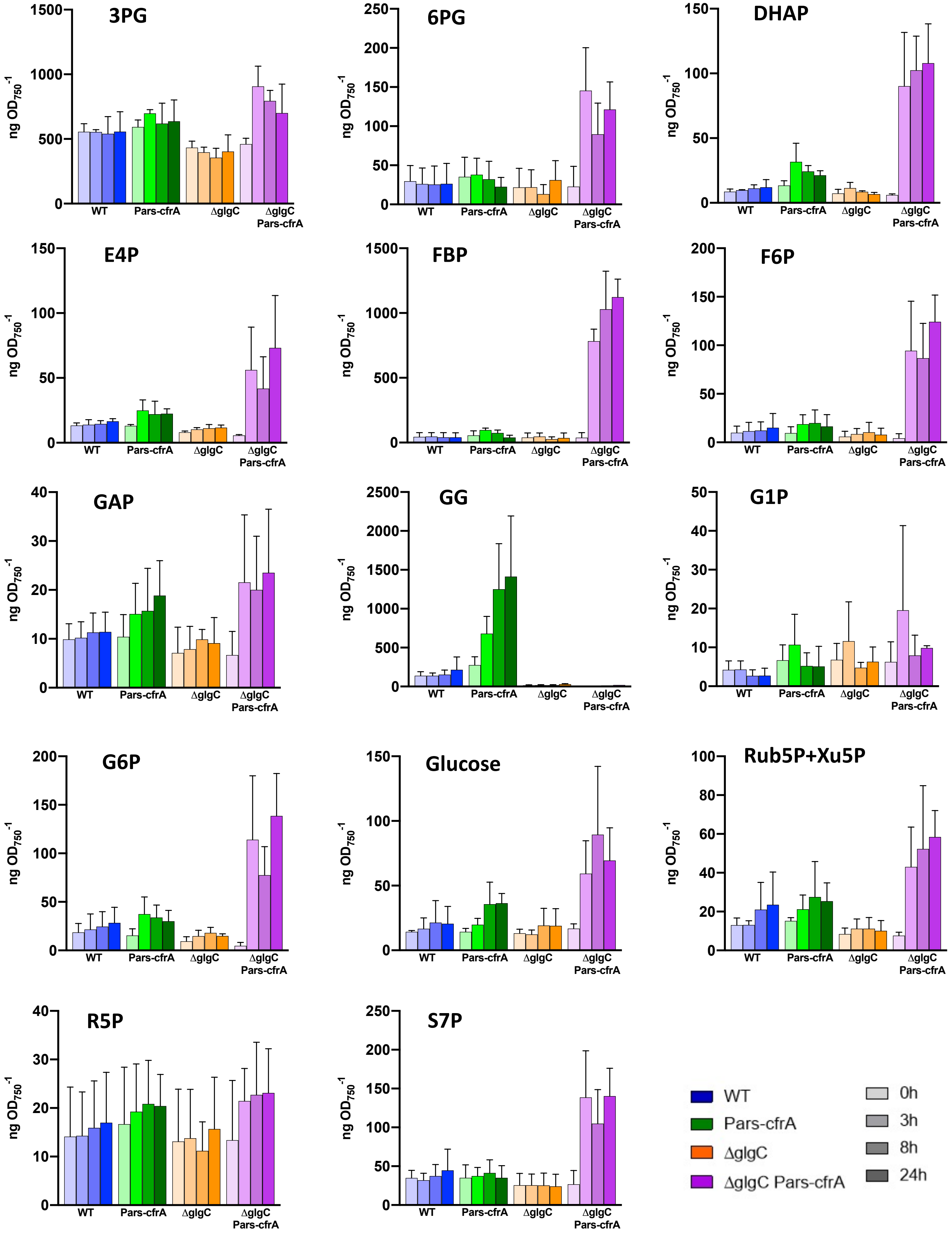

Supplemental Figure S3. Time-course LC-MS analysis of all analyzed carbohydrates as a function of *cfrA* expression. Each bar represents the metabolite level at a certain time, determined from three independent experiments with three technical replicates at each point. The error bars represent the SD of the combined data.

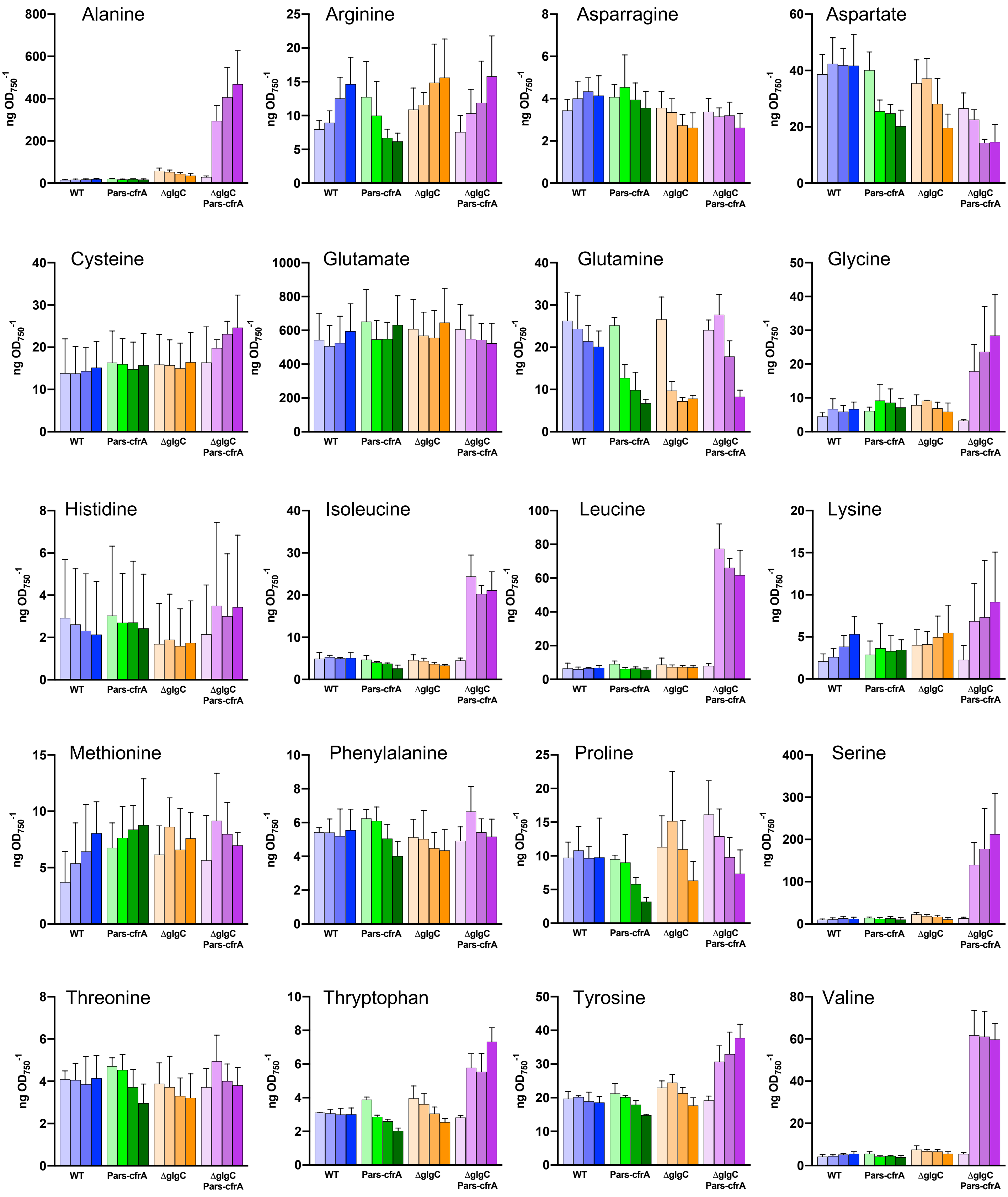

Supplemental Figure S4. Time-course LC-MS analysis of all analyzed amino-acids as a function of *cfrA* expression. Each bar represents the metabolite level at a certain time, determined from three independent experiments with three technical replicates at each point. The error bars represent the SD of the combined data. The color legend of the strains and temporal kinetics is the same as that of Supplemental Figure S3.

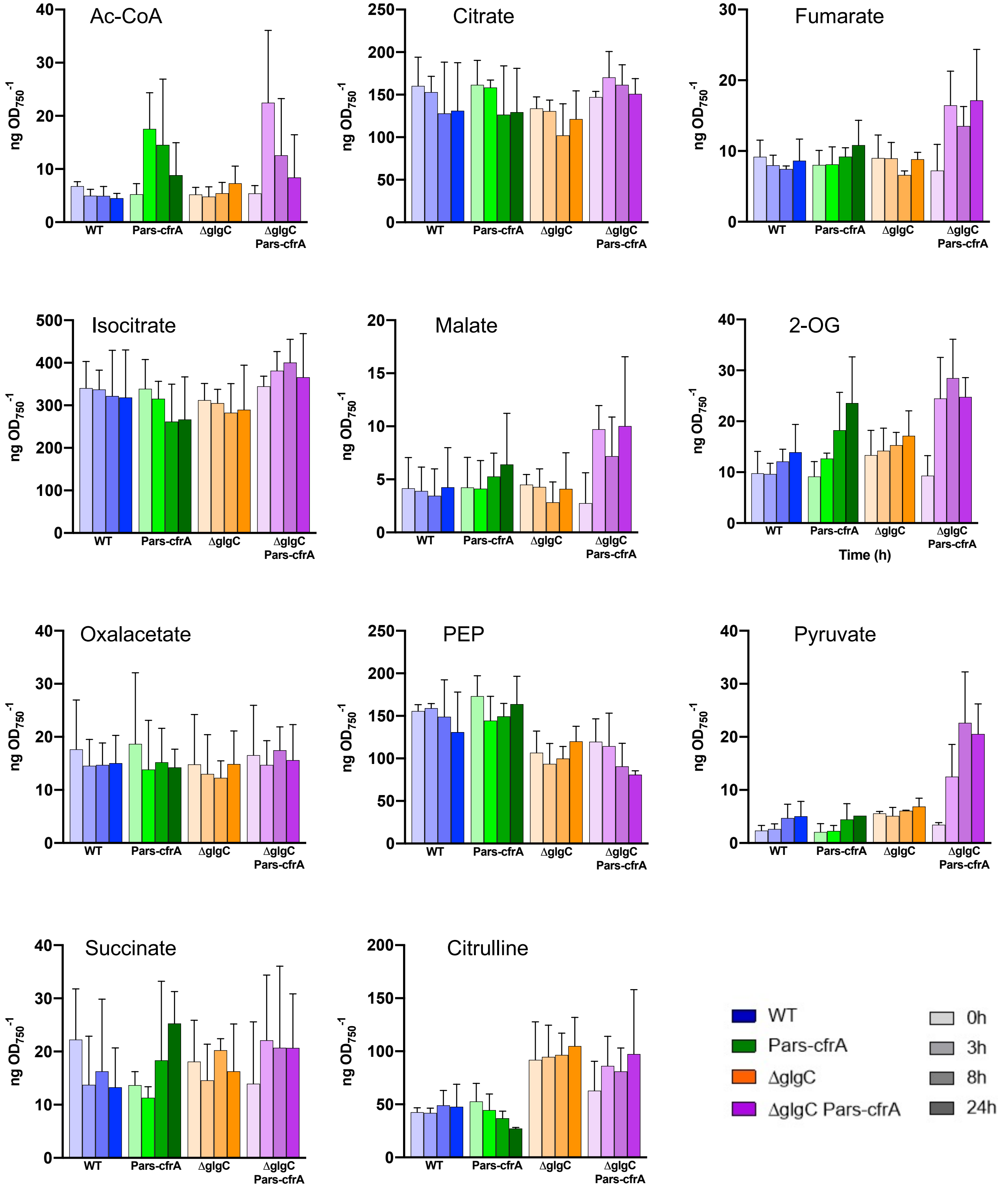

Supplemental Figure S5. Time-course LC-MS analysis of some metabolic intermediaries analyzed as a function of *cfrA* expression. Each bar represents the metabolite level at a certain time, determined from three independent experiments with three technical replicates at each point. The error bars represent the SD of the combined data.

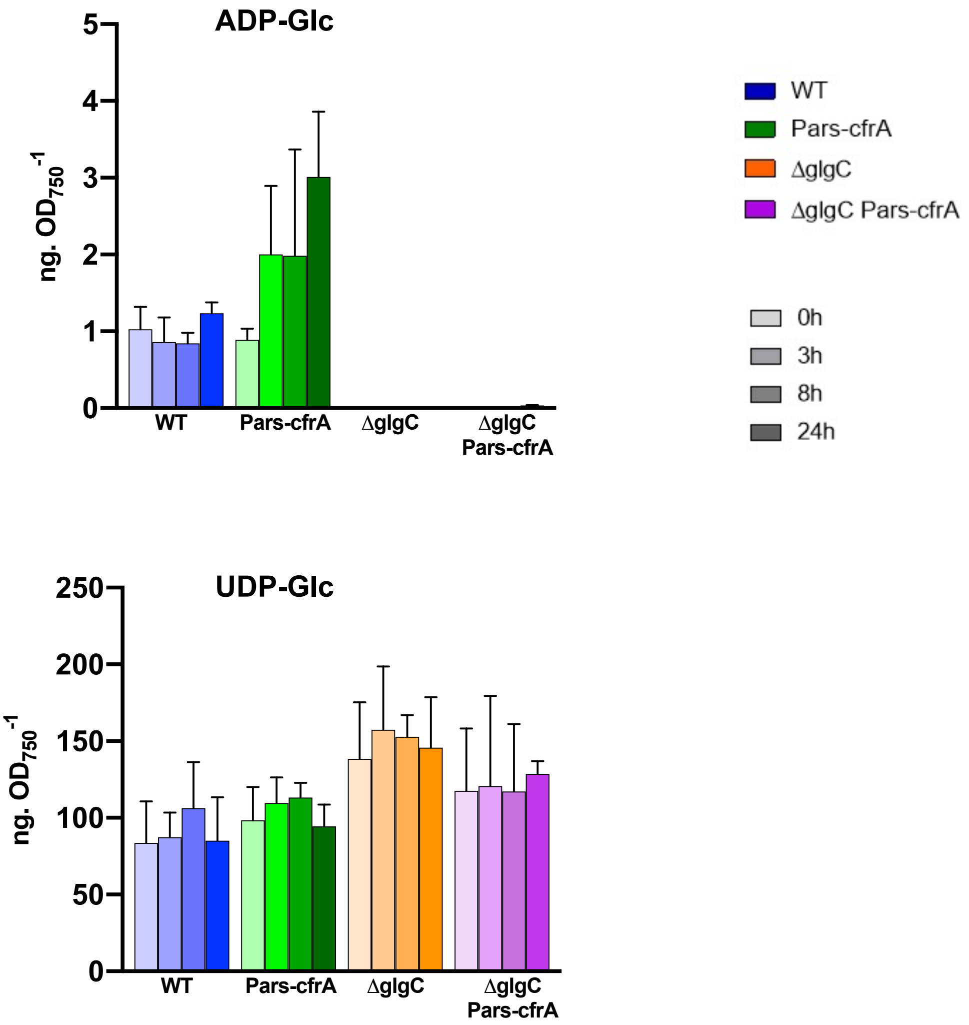

Supplemental Figure S6. Time-course LC-MS analysis of ADP-glucose (ADP-Glc) and UDP-glucose (UDP-Glc) as a function of *cfrA* expression. Each bar represents the metabolite level at a certain time, determined from three independent experiments with three technical replicates at each point. The error bars represent the SD of the combined data.
